# Ultrasound estimated subcutaneous and visceral adipose tissue thicknesses and risk of pre-eclampsia

**DOI:** 10.1038/s41598-021-02208-z

**Published:** 2021-11-23

**Authors:** Heidrun Pétursdóttir Maack, Inger Sundström Poromaa, Linda Lindström, Ajlana Mulic-Lutvica, Katja Junus, Anna-Karin Wikström

**Affiliations:** Department of Women’s and Children’s Health, Uppsala University, 751 85 Uppsala, Sweden

**Keywords:** Diseases, Medical research, Risk factors

## Abstract

Early identification of high-risk pregnancies enables identification of those who would benefit from aspirin prophylaxis and increased surveillance for pre-eclampsia. A high body mass index (BMI) is a well-known predictor for pre-eclampsia. However, if abdominal adipose tissue distribution is associated with pre-eclampsia is limited investigated. Subcutaneous adipose tissue (SAT) thickness and visceral adipose tissue (VAT) thickness were measured by ultrasound on 3777 women at around 18 gestational weeks. SAT thickness was measured from the skin to linea alba and VAT from linea alba to the anterior aortic wall. The risk of developing pre-eclampsia (de novo hypertension at ≥ 20 gestational weeks in combination with proteinuria) was evaluated by logistic regression and expressed as odds ratio (OR) with 95% confidence intervals (CI). The risk of pre-eclampsia increased by 79% for every cm in SAT thickness (OR 1.79; 95% CI 1.48–2.17) and by 23% for every cm VAT thickness (OR 1.23; 95% CI 1.11–1.35). After adjustment for maternal age, parity, BMI, smoking and country of birth, the association between SAT thickness and pre-eclampsia remained (AOR 1.35; 95% CI 1.02–1.79). Greater SAT thickness measured with second trimester ultrasound is associated with increased risk of developing pre-eclampsia. The measurement may improve prediction models for pre-eclampsia.

## Introduction

Pre-eclampsia complicates about 3–8% of pregnancies^1^ and can entail devastating complications for both mother and foetus^2^. Identification of women with high risk for pre-eclampsia in early pregnancy enables aspirin prophylaxis and individual surveillance, which may lead to earlier detection of the disorder^3,4^. Many risk factors for developing pre-eclampsia have been described, one of those is obesity^2,5,6^. Currently, about one-third of women of reproductive age in the USA are obese^7^ and the global obesity epidemic is a target for the United Nations third Sustainable Development Goal^8^. Identifying those with the highest risk of pregnancy complications among obese women is therefore important.

The obesity definition is based on body mass index (BMI), which does not consider muscle mass and adipose tissue distribution. Studies on adipose tissue distribution in the general population show that both subcutaneous adipose tissue (SAT) and visceral adipose tissue (VAT) are associated with metabolic risk factors such as blood pressure, fasting plasma glucose, and high-density lipoprotein cholesterol^9,10^. However, some studies state that VAT is a stronger indicator than SAT^11^. In the general population VAT/SAT ratio has been shown to be a predictor for cardiac events.

Several methods can be used to estimate adipose distribution. Of those, computed tomography (CT) and magnetic resonance imaging (MRI) seem to provide the best estimation of visceral fat^12,13^. Measurement of VAT and SAT with ultrasonography is a potential alternative; it is non-invasive, considered as low-risk for both mother and foetus, and compares well with the gold standard CT measurement^14,15^.

Ultrasound measurements of abdominal adipose tissue distribution during pregnancy have only been performed in small series^16–22^. To our knowledge, only three studies have investigated the association of abdominal adipose tissue distribution estimated by ultrasound and risk of hypertensive disorders in pregnancy^16,22,23^, whereof only one studied pre-eclampsia specifically^23^ and all study populations were comparatively small.

Our aim in this population-based study of around 4000 women was to investigate if the ultrasonically assessed adipose tissue distribution could be used as a predictor for developing pre-eclampsia later during pregnancy.

## Methods

### Study population

For this prospective cohort study we included women who did their routine second trimester foetal ultrasound scan at Uppsala University hospital between January 2015 and January 2019. We obtained ethical approval to implement SAT and VAT measurements in conjunction with the ultrasound examination and to evaluate the usefulness of the measures by linkage to standardized hospital electronic medical records on maternal, obstetric, and perinatal health care. Following linkage, the study population database was anonymized. The Regional Ethical Review Board in Uppsala approved the study on September 24th 2014 (Dnr: 2014/353). All research was performed in accordance with relevant national and international guidelines. Informed consent was waived by the Swedish Ethical Review Authority (Dnr: 2019-00391). As the study was register-based with anonymized data this was not required. In general, large register-based studies in Sweden do not require informed consent. Ludvigsson et al.^24^ explained this, that as long as a register-based study is deemed ethical by committee, it is assumed that the participants do not object to the research.

**Figure 1:**
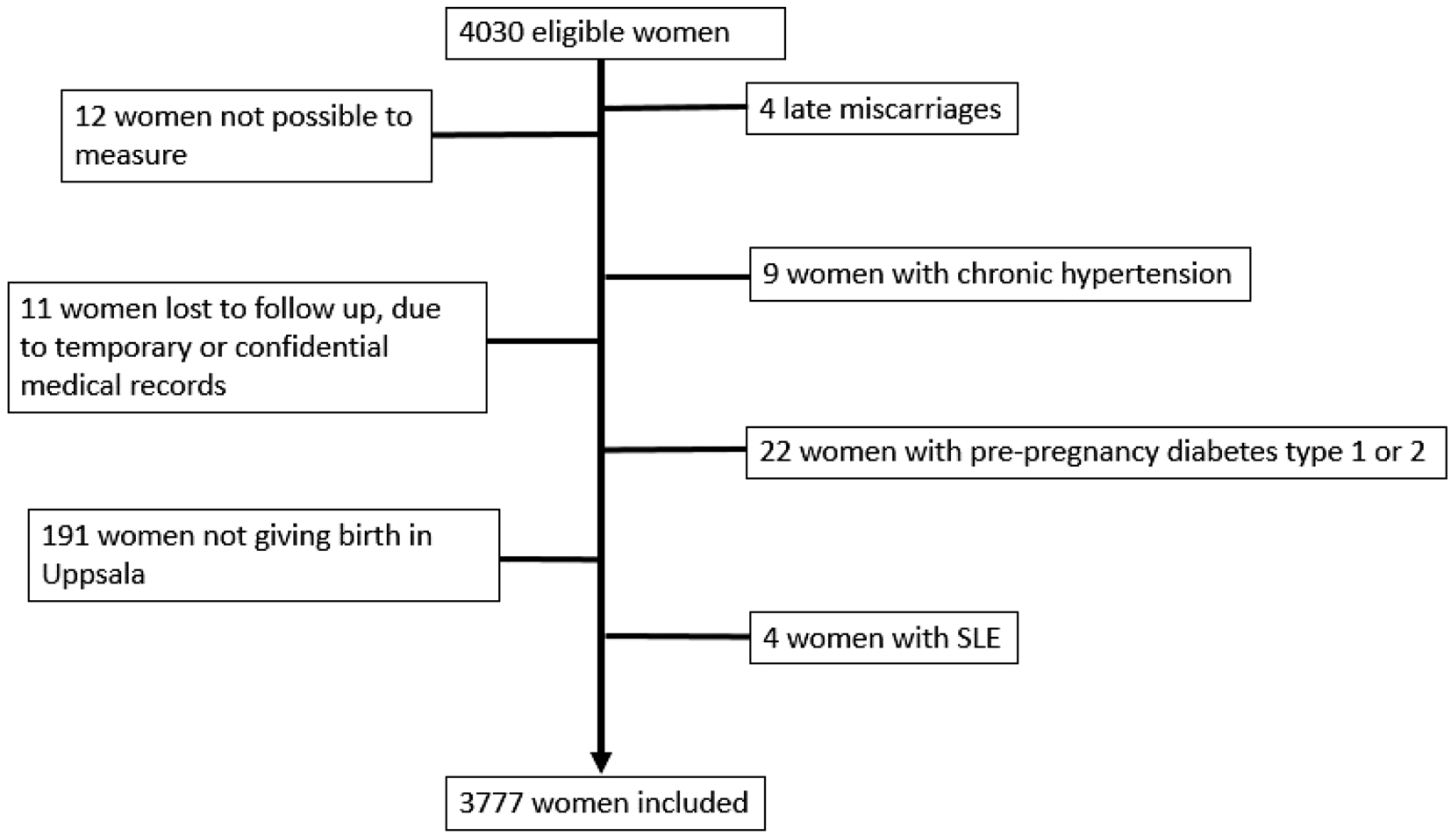
Flow chart of the study population.

All pregnant women in Sweden are invited to the routine second trimester foetal ultrasound scan at 17–19 weeks of gestation. In Uppsala County, all obstetric ultrasound scans are performed at Uppsala University Hospital, which also holds the only delivery ward within the county. Hence, the women included represent a population-based sample. Women were eligible for the study if they had an appointment with one of the certified obstetric ultra-sonographers who had been trained on the SAT and VAT measurement, see further below, and if they accepted participation (n = 4030). We excluded women where the measurements were not possible to perform because of lack of visualisation of the rectus musculature (n = 12), women we could not link with the hospital electronic medical records because of temporary or confidential medical records (n = 11), women with late miscarriage (n = 4), and 191 women who moved during the study time or did not give birth in Uppsala. Further, we excluded women with chronic hypertension (n = 9), pre-pregnancy diabetes type 1 or 2 (n = 22) or systemic lupus erythematosus (n = 4), all known risk factors for pre-eclampsia. The final study population consisted of 3777 pregnant women, Fig. 1.

### Exposure

**Figure 2:**
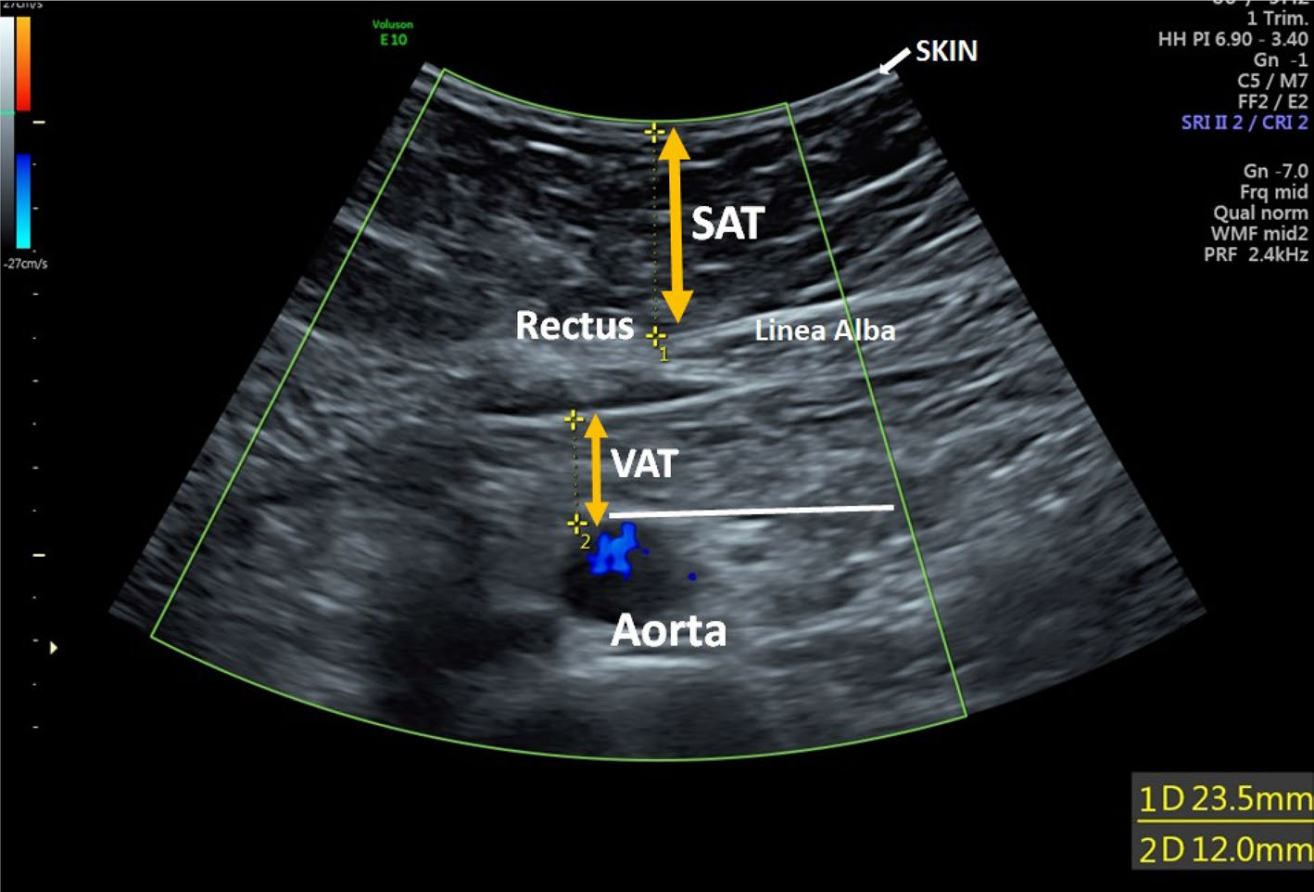
Adipose tissue measurement taken 10 cm above the level of the umbilicus. The subcutaneous adipose tissue (SAT) thickness measured from the inner border of the skin down to line alba. The visceral adipose tissue (VAT) thickness measured from the posterior edge of the line alba to the anterior aortic wall.

The SAT and VAT thickness measures were performed by certified obstetric ultra-sonographers, using Voluson E6, E8 or E10 GE ultrasound machines (GE Healthcare, Zipf, Austria). The certified obstetric ultra-sonographers had been specifically trained to perform the adipose tissue measurements according to the method described by Armellini and colleagues^25^. Additional training sessions were carried out during the study period to maximize the quality of the scans. The ultrasound measures were taken 10 cm above the level of the umbilicus. The SAT thickness was measured from the inner border of the skin down to linea alba, the midline connecting part between the abdominal rectus musculature. The VAT thickness was measured from the posterior edge of the linea alba to the anterior aortic wall, Fig. 2. All the measurements were done with gentle pressure by the ultrasound probe to avoid underestimation of the measurements. An appropriate round transverse section of the aorta was used as a good control point, i.e. if an oval aorta was visualised during the measurement, the pressure of the ultrasound probe was reduced until a round transverse section was visualised. The SAT and VAT thickness measurements were documented in millimetres, and ultrasound images were saved on VeiwPoint 6, version 6.10 (GE Healthcare, Chicago, IL, URL: ViewPoint 6 for OB/GYN | GE Healthcare (United States). The intraclass correlation coefficient (ICC) of the inter-examiner variation in SAT measurements was 0.85, and 0.83 in VAT measurements, both indicating good reliability^26^. The VAT and SAT thickness ratio (VAT/SAT) was then calculated from the measurements. We chose to estimate the increased risk of pre-eclampsia for every centimetre (cm) increase in adipose tissue thickness instead of every millimetre (mm) increase. Reporting in mm overstates the exactness of the ultrasound measurement and the use of cm produces a result that is understandable and communicable to physicians and patients.

### Main outcome

The main outcome was pre-eclampsia defined as de novo hypertension after 20 weeks of gestation in combination with proteinuria, which was the clinical definition of the disorder in Sweden during the study period. Hypertension was defined as systolic blood pressure of ≥ 140 mmHg and/or diastolic blood pressure of ≥ 90 mmHg measured on two subsequent occasions, at least 6 h apart. Proteinuria was defined as ≥ 2 + on a dipstick or ≥ 300 mg/24 h in a urine collection. During the study period, women with one of the following major risk factors were recommended 75 mg aspirin from gestational week 12: history of severe or preterm pre-eclampsia in earlier pregnancy, chronic kidney disease, diabetes mellitus with known vascular complications or autoimmune disease such as systemic lupus erythematosus with anticardiolipin antibodies.

### Maternal demographics and clinical outcomes

Information about maternal demographic and clinical variables, pregnancy complications and perinatal outcome was obtained via linkage with the standardized hospital electronic antenatal, obstetric and neonatal medical records. Demographic data collected at the first antenatal visit included: maternal age, parity, early pregnancy BMI, self-reported smoking habits (yes/no), country of birth (born inside EU/born outside EU), pregnancy conceived by assisted reproduction, and present comorbid diseases. The women’s weight in kg and height in cm were measured and recorded at the first antenatal booking, and BMI (kg/m^2^) was calculated. The women were classified as underweight (BMI < 18.5 kg/m^2^), normal weight (BMI 18.5–24.99 kg/m^2^), overweight (BMI 25–2.99 kg/m^2^) or obese (BMI > 30 kg/m^2^). Comorbid diseases included rheumatic diseases (yes/no) and inflammatory disorders (yes/no). Clinical variables and outcomes included gestational length in days at delivery. Gestational length was categorized into preterm (< 37 gestational weeks) or term (≥ 37 gestational weeks) birth. Birthweight was measured in grams. Further, we recorded if the infant was born small for gestational age (SGA), defined as birth weight below the third percentile, according to the Swedish sex-specific reference curves for gestational age^27^, or large for gestational age (LGA) as above the 97th percentile.

### Statistics

Demographic and clinical variables, including SAT and VAT thickness, in women who developed and not developed pre-eclampsia, were compared by use of independent t-tests or Chi-square test. The correlation between BMI and SAT and VAT thickness, respectively, were calculated with Pearson’s t-tailed correlation test.

We determined the risk of developing pre-eclampsia by use of logistic regression analysis, with SAT and VAT thickness as well as VAT/SAT ratio expressed in centimetres as predictors. We estimated unadjusted and adjusted odds ratios (OR) with their corresponding 95% confidence intervals (CI). To identify possible confounders, we drew a directed acyclic graph (DAG), supplementary Fig. 1^28^. We then used the minimal sufficient adjustment sets for estimating the direct effect of abdominal adipose tissue thickness on pre-eclampsia. According to the DAG we included age (as a continuous variable), BMI (as a continuous variable), country of origin (born in the European Union or born outside the European Union), self-reported smoking at the beginning of pregnancy (yes/no) and parity (as a continuous variable) in our adjusted model. All of these variables are known risk factors for developing pre-eclampsia^3^, except smoking which is inversely associated with pre-eclampsia^29^, and also related to increased abdominal adipose tissue^30–33^. For all the statistical analyses, we used IBM SPSS Statistics, version 25.

## Results

### Description of study population

The study population is described in Table 1. Among the 3777 women in the study population, 138 (3.7%) women developed pre-eclampsia. Women with pre-eclampsia were more often nulliparous and had a higher BMI in early pregnancy compared to women without pre-eclampsia. There was no difference in rates of assisted reproduction or rheumatic or inflammatory chronic disorder in women with and without pre-eclampsia. The gestational length at delivery was shorter for women with pre-eclampsia, and they gave birth to infants with lower birth weights. The risk for preterm birth was higher among women with pre-eclampsia, of whom 15.9% gave birth before 37 gestational weeks, indicating preterm pre-eclampsia. Women with pre-eclampsia gave birth to SGA infants more often compared to women without pre-eclampsia (6.8% vs. 0.8%), but no difference was found for giving birth to LGA infants. 55 women (1.5%) were treated with prophylactic aspirin. Of those, 9 women were nulliparous, and 6 of the women treated with aspirin developed pre-eclampsia.

**Table 1:** Demographic and clinical variables of the study population.

	Pre-eclampsia (n = 138)	Non-pre-eclampsia (n = 3639)	*p*-value
Maternal age, years	30.3 ± 4.8	30.6 ± 4.8	0.389
Nulliparous	86 (62.3%)	1575 (43.3%)	< 0.001
**First trimester BMI, kg/m**^**2**^	27.5 ± 6.5	25.0 ± 4.9	< 0.001
< 18.5 (underweight)	4 (2.9%)	82 (2.3%)	
18.5–24.9 (normal weight)	57 (41.3%)	2076 (57%)	
25.0–29.9 (overweight)	37 (26.8%)	899 (24.7%)	
≥ 30.0 (obese)	40 (29%)	588 (15.3%)	
Smoking	4 (2.9%)	77 (2.1%)	0.533
Born outside EU	22 (15.9%)	503 (13.8%)	0.453
Prophylactic aspirin treatment	6 (4.3%)	49 (1.3%)	0.004
Assisted reproduction	4 (2.9%)	183 (5%)	0.257
Rheumatic disorder	1 (0.7%)	24 (0.7%)	0.926
Inflammatory disorder	1 (0.7%)	28 (0.8%)	0.953
Gestational length at birth, days	271 ± 19	278 ± 11	< 0.001
Preterm birth	22 (15.9%)	140 (3.8%)	< 0.001
Birth weight, grams	3263 ± 754	3577 ± 509	< 0.001
SGA	9 (6.8%)	27 (0.8%)	< 0.001
LGA	7 (5.3%)	136 (3.8%)	0.247

Data presented as mean ± SD or n (%). BMI = body mass index; SGA = small for gestational age (birth weight below the third percentile); LGA = large for gestational age (birth weight above 97th percentile). Statistical analyses by independent t-test or Chi-square test.

### Correlation between BMI and adipose tissue thickness

**Figure 3:**
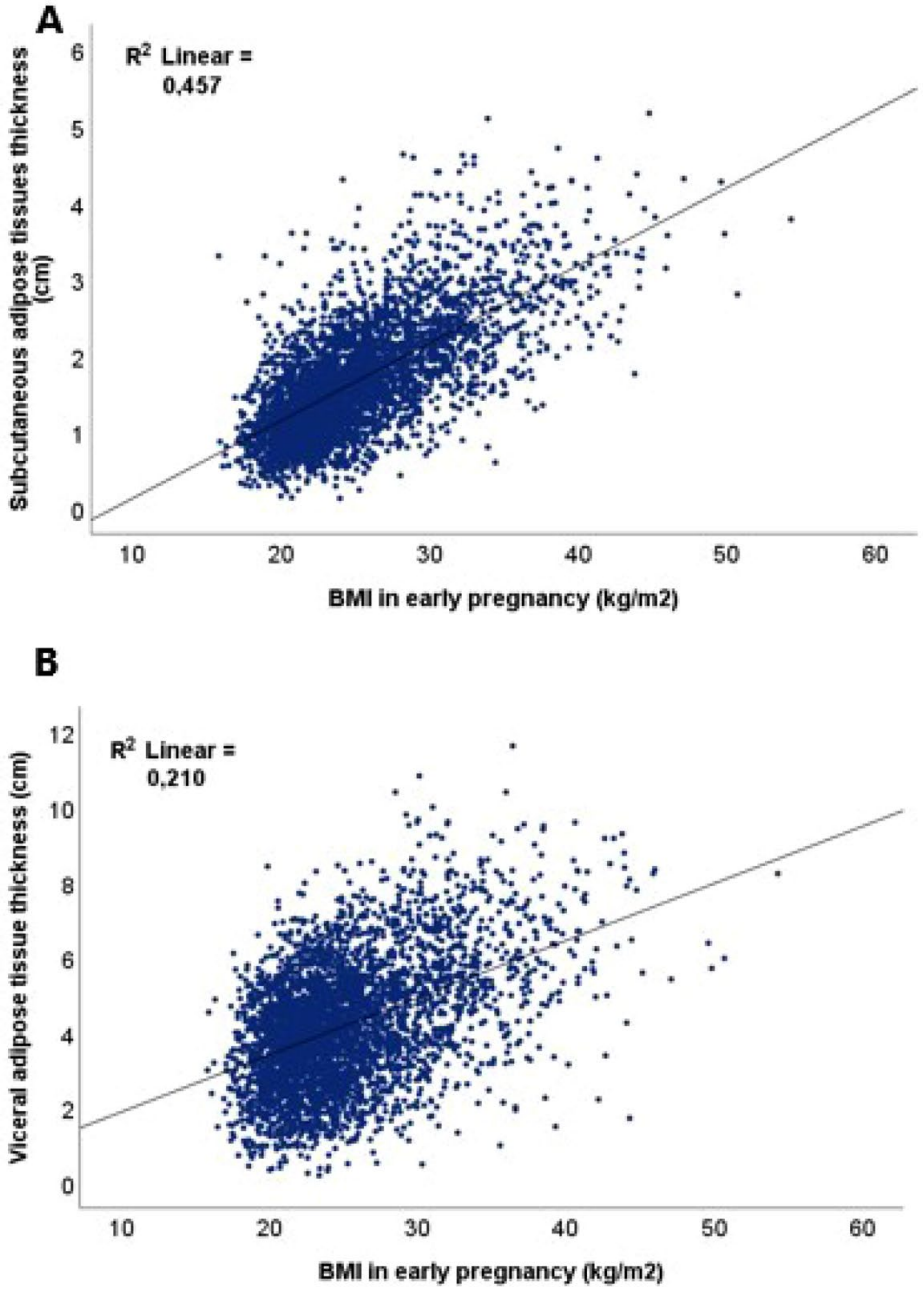
Correlation between body mass index (BMI) and subcutaneous adipose tissue (SAT) thickness (**A**) and visceral adipose tissue (VAT) thicknesses (**B**).

BMI was highly and positively correlated with SAT thickness, *r* = 0.68, *p* < 0.001, Fig. 3A. Similarly, we found a positive correlation between BMI and VAT thickness, *r* = 0.46, *p* < 0.001, Fig. 3B.

### BMI and its association to pre-eclampsia

Women who developed pre-eclampsia had higher BMI than those without pre-eclampsia, 27.5 ± 6.5 kg/m^2^ vs. 25.0 ± 4.9 kg/m^2^. Women with higher BMI had an increased risk of developing pre-eclampsia, OR 1.08 (95% CI 1.06–1.12), *p* < 0.001.

### Adipose tissue thickness in women with or without pre-eclampsia

Women with pre-eclampsia had a SAT thickness of 2.04 ± 0.89 cm, whereas the corresponding measure in women without pre-eclampsia was 1.65 ± 0.73 cm, *p* < 0.001. Similarly, greater VAT thickness was noted in women with pre-eclampsia 4.75 ± 1.79 cm, compared to women without pre-eclampsia 4.16 ± 1.63 cm, *p* < 0.001.

Women with greater SAT thickness more often developed pre-eclampsia, OR 1.79 (95% CI 1.48–2.17), i.e. with every centimetre increase in SAT thickness, the risk of pre-eclampsia increased by 79%. Greater VAT thickness was also associated with increased risk of developing pre-eclampsia, OR 1.23 (95% CI 1.11–1.35), suggesting that every extra centimetre VAT thickness increased the risk by 23%. After adjustment for confounders, the association between SAT thickness and pre-eclampsia remained, AOR 1.35 (95% CI 1.02–1.79), but no longer remained significant for VAT thickness; AOR 1.11 (95% CI 0.99–1.24), Table 2.

**Table 2:** Subcutaneous adipose tissue (SAT) thickness and visceral adipose tissue (VAT) thickness in women later developing and not developing pre-eclampsia.

	Pre-eclampsia (n = 138)	Non-pre-eclampsia (n = 3639)	OR (95% CI)	*p*	AOR (95% CI)^a^	*p*
Subcutaneous adipose tissue (cm)	2.04 ± 0.89	1.65 ± 0.73	1.79 (1.48–2.17)	**0.000**	1.35 (1.02–1.79)	**0.037**
Visceral adipose tissue (cm)	4.75 ± 1.79	4.16 ± 1.63	1.23 (1.11–1.35)	**0.000**	1.11 (0.99–1.24)	0.080
VAT/SAT ratio	2.69 ± 1.60	2.94 ± 1.71	0.90 (0.80–1.02)	0.090	0.99 (0.88–1.12)	0.923

Odds ratio (OR) illustrates increased risk by every centimetre increase in tissue thickness.

Data missing on SAT measurement on 2 patients and on VAT measurement on 6 patients.

*CI* confidence Interval, *VAT* visceral adipose tissue, *SAT* subcutaneous adipose tissue.

^a^Adjusted for maternal age, parity, BMI, smoking and country of birth.

When the analyses were repeated after excluding all women treated with prophylactic aspirin, the estimates were only marginally changed for both SAT and VAT. The association between SAT thickness and risk of pre-eclampsia was OR 1.80 (95% CI 1.48–2.19), with AOR 1.34 (95% CI 1.00–1.79), including adjustment for BMI. The corresponding associations between VAT thickness and pre-eclampsia were OR 1.21 (95% CI 1.09–1.33), and AOR 1.09 (95% CI 0.97–1.22).

Women with pre-eclampsia had a VAT/SAT ratio of 2.69 ± 1.60, whereas the corresponding ratio in women without pre-eclampsia was 2.94 ± 1.71. Greater VAT/SAT ratio was not associated with increased risk of developing pre-eclampsia, OR 0.90 (95% CI 0.80–1.02), AOR 0.99 (0.88–1.12).

## Discussion

Our study shows that increased abdominal adipose tissue thickness in pregnancy is associated with development of pre-eclampsia. This association was stronger with SAT than VAT thickness, and the association remained significant after adjustment for confounders, including BMI.

To our knowledge, there are only three earlier studies that have measured SAT or VAT thickness during the first half of pregnancy and estimated the association with risk of hypertensive disorders in pregnancy. Altogether, our results are in agreement with these reports. Only one has studied pre-eclampsia specifically^23^; it included 463 pregnant women, and only VAT was measured. Their results were similar to ours, with significant association between VAT and pre-eclampsia only in unadjusted models. The studies of Nassr et al. and Kennedy et al. both studied hypertensive disorders in pregnancy as an outcome. Nassr et al.^21^ used another ultrasound method of estimating SAT and VAT compared to our study and included only 389 women. They found that both SAT and VAT were associated with the development of hypertensive disorders in pregnancy, using specific cut-off values for SAT and VAT. In our study, we did not find an association between VAT and pre-eclampsia. This discrepancy might depend on the different methods used to estimate VAT and to difference in risk estimation as we used continuous measurement on VAT instead of cut-off values. In the study of Nassr et al., most women developed gestational hypertension, not pre-eclampsia, two disorders with partly different pathophysiology and clinical outcomes^34,35^. Our results are consistent with the previous report of Kennedy et al.^16^, including 1510 pregnant women, showing that SAT thickness is a BMI-independent predictor for pregnancy hypertensive disease. Compared to all three former reports, our study population was more homogenous since we excluded women with twin pregnancies and some chronic disorders known as risk factors for pre-eclampsia.

To our knowledge, there are no studies on VAT/SAT ratio and association with pre-eclampsia. In our study VAT/SAT ratio was not associated with increased risk of pre-eclampsia, indicating that the ratio between different kinds of adipose tissue is not of great relevance, but rather that the adipose tissue thickness itself is the driving factor.

The exact causal pathway between obesity and pre-eclampsia is poorly understood, but both have several pathophysiological alterations in common, such as oxidative stress, inflammation and endothelial dysfunction^6^. The literature suggests that metabolic abnormalities may be a large part of the causal pathway between obesity and pre-eclampsia^36^. Since both SAT and VAT thickness are correlated with metabolic risk factors^11^, it is not unlikely that central obesity in early pregnancy can be a part of the link between obesity and risk of developing pre-eclampsia. We had anticipated that VAT thickness would be a better predictor for pre-eclampsia than SAT thickness, as studies in non-pregnant populations indicate that VAT is a stronger indicator for metabolic risk factors^11^. In addition, during early pregnancy, VAT seems to correlate stronger to the same metabolic risk factors than SAT^21^. Ageing is associated with an increase in fat deposition, especially in the visceral area^37^. As pregnant women are comparatively young, deposition to visceral areas may yet not have started. This might explain why SAT thickness seemed to carry stronger association with pre-eclampsia than VAT thickness.

The main strength of our study is the large homogenous study population of 3777 pregnant women and the population-based design. This is, to our knowledge, one of the largest studies with adipose tissue measurements during pregnancy. Further, in comparison with previous efforts, we specifically studied pre-eclampsia. We also measured both SAT and VAT in the same study population and estimated the ratio between the estimates, which has not been done before in pregnant women. We used a validated method to estimate SAT and VAT, a method that is easy to implement in routine ultrasound scans during the first half of pregnancy.

Several limitations in our study are important to mention. Although our study population is relatively large, only 138 women developed pre-eclampsia, of whom 22 developed preterm pre-eclampsia. Therefore, we could not reach power to evaluate the association between adipose distribution in early and late pre-eclampsia. Another major limitation is that we performed the adipose tissue measurement at gestational weeks 17–19, instead of earlier in pregnancy. However it has been shown that abdominal adipose tissue distribution changes in the third trimester when compared to both first and second trimester, but those changes are not seen between first and second trimester^38^. According to our results, the variation in SAT and VAT measurement for any given BMI was greater than expected. Armellini et al.^14^ showed that the correlation between ultrasound and CT was in the range of 0.68–0-74. However, the gold standard methods for measuring abdominal adipose tissue are not acceptable during pregnancy for safety reasons. The variability can be partly explained by measurement errors, although all ultra-sonographers performing the measurements were certified and highly skilled. A possible explanation for this variation is a difference in body composition of the young study population, where some individuals have a high proportion of muscle mass, and some a greater proportion of adipose tissue, which BMI does not accurately reflect.

Today prediction and prevention of pre-eclampsia have gained great attention, both in research and clinically. Aspirin treatment starting at gestational week 16 or earlier in women at high risk for preterm pre-eclampsia, reduces the risk for preterm pre-eclampsia^39,40^, but not term pre-eclampsia^41^. The greatest challenge is to identify women who will benefit from aspirin treatment, without over-treating. It is therefore of great importance that the identification of high-risk women for pre-eclampsia is predicted before the end of the first trimester. We also did not have information about history of pre-eclampsia in earlier pregnancies, which is one of the strongest risk factors for developing pre-eclampsia in multiparous women^42^. However, women with a history of severe or early pre-eclampsia were routinely recommended aspirin. In a sensitivity analysis excluding women on aspirin prophylaxis, the association between SAT and risk of pre-eclampsia remained.

An important issue in need of further research is aspirin prophylaxis in the obese population for pre-eclampsia prevention. Evidence suggests that aspirin may be less effective in the general obese population, and there are several potential mechanisms behind this^43^. Finneran et.al. published 2019 a study on the association of obesity on platelet-derived thromboxane inhibition in high-risk women treated with low-dose aspirin. Their data suggested that an increase in aspirin dosing or frequency may be necessary in the obese population^44^. Other studies, such as the one by Cantu and collage, have shown no difference in the efficiency of low-dose aspirin between obese and non-obese women^45^. Further research is needed to evaluate if SAT and VAT measurements would contribute to risk assessment model in order to identify obese women who would benefit from prophylactic treatment with aspirin.

High BMI is a common risk factor in all prediction methods for pre-eclampsia, and our results show that an increase of BMI by one unit increases the risk of pre-eclampsia by 8%. Further studies are needed to investigate the potential role of SAT and VAT thickness in the first- or early second trimester. Prediction models for pre-eclampsia used today need to improve their prediction performance. Adding SAT measurement to such a model might be the next step. As most prediction models for pre-eclampsia today address risk factors as continuous measurements, we find it important to address adipose tissue measurements as a continuous variable. The method used to estimate adipose tissue thickness has the potential to be effective, quick and highly cost-effective.

In conclusion, our study shows that greater SAT thickness measured with ultrasound in early second trimester is associated with an increased risk of developing pre-eclampsia later in pregnancy.

## Supplementary Information

Supplementary Figure 1.
