# Supplementary figures and images for: Ultrasound estimated subcutaneous and visceral adipose tissue thicknesses and risk of pre-eclampsia
Source: Sci Rep. 2021 Nov 23;11:22740. doi: 10.1038/s41598-021-02208-z (PMC8611080; doi:10.1038/s41598-021-02208-z)

Supplementary Figure 1. DAG for identifying possible confounders


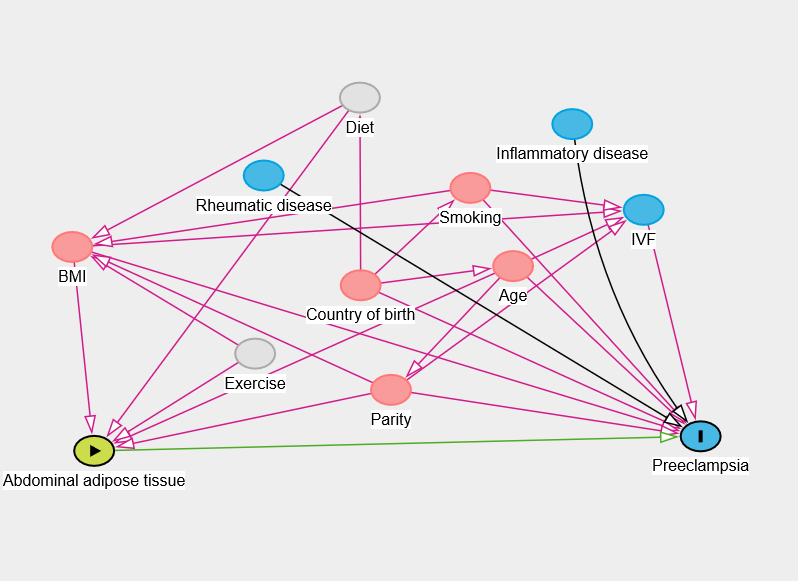

Supplement: Supplementary file 1 — Supplementary Figure 1. [file 41598_2021_2208_MOESM1_ESM.docx]
